# Efforts towards a precision medicine approach in juvenile idiopathic arthritis

**DOI:** 10.1093/rheumatology/keag402

**Published:** 2026-08-06

**Authors:** Christine Chew, Gareth W Jones, Adam P Croft, Lucy R Wedderburn, Athimalaipet V Ramanan

**Affiliations:** School of Cellular and Molecular Medicine, University of Bristol, Bristol, UK; Paediatric Rheumatology, Bristol Royal Hospital for Children, Bristol, UK; Paediatric Rheumatology, Great Ormond Street Hospital, London, UK; School of Cellular and Molecular Medicine, University of Bristol, Bristol, UK; Rheumatology Research Group, Institute of Inflammation and Ageing, University of Birmingham, Birmingham, UK; National Institute of Health Research Biomedical Research Centre, University Hospitals Birmingham NHS Foundation Trust, Birmingham, UK; UCL GOS Institute of Child Health, University College London, London, UK; Centre for Adolescent Rheumatology at UCL, UCLH and GOSH, UCL, London, UK; National Institute of Health Research Biomedical Research Centre at GOSH London UK, Great Ormond Street Hospital, London, UK; Paediatric Rheumatology, Bristol Royal Hospital for Children, Bristol, UK; Translational Health Sciences, University of Bristol, Bristol, UK

**Keywords:** juvenile idiopathic arthritis, immunology, inflammation, biomarkers, synovium, microarrays/gene expression profiling/transcriptome, bioinformatics, proteomics, functional genomics, biological therapies

## Abstract

Juvenile idiopathic arthritis (JIA) is the commonest group of childhood arthritides. Despite the availability of advanced therapeutics, many children and young people (CYP) with JIA experience disease flares, and in some, chronic joint damage. Tailoring treatment based on unique biological profiles would benefit CYP with JIA given their variable clinical presentation and disease course. To date, biomarkers to predict treatment response are lacking. With advances in single cell technologies, we are now able to profile the genes and proteins of target tissues at unprecedented resolution to define the biological basis of disease and guide novel treatment approaches. The complex analyses and combination of biological and clinical outcome data from large datasets across disease phenotypes have become possible with the development of computational and machine learning methods. Here, we summarize the strategies to integrate data through multimodal based approaches to maximize precision medicine and research priorities for CYP with JIA.

Rheumatology key messages

- A significant proportion of CYP with JIA have persistent articular and extra-articular disease despite the breadth of therapies available.
- Clinical and laboratory measures do not reliably predict treatment response or disease activity.
- Evolving single cell technologies and bioinformatics may enable the integration of synovial cellular, molecular, and structural profiles with tissue-specific biomarkers.

## Introduction

Juvenile idiopathic arthritis (JIA) comprises a heterogeneous group of conditions used to describe arthritis lasting at least 6 weeks and starting before 16 years of age [1]. In the UK, between 1 in 1000 and 1 in 2000 children and young people (CYP) are affected by JIA [2–4]. Treatment of early JIA, during the so called ‘window of opportunity’ [5], targets a period shortly after disease onset and aims to prevent joint and tissue damage, reduce extra-articular risk, preserve function, and increase the likelihood of achieving clinical remission without the global drug burden throughout life [6, 7].

Long-term outcomes of JIA have seen remarkable improvement with the availability of advanced targeted therapies. In most individuals with JIA, non-steroidal anti-inflammatory drugs, intra-articular joint injection and/or methotrexate as the first line conventional synthetic (cs) disease-modifying antirheumatic drug (DMARD) are used [8, 9]. Methotrexate is the first drug of choice in combination with folic acid, despite its being effective or tolerated in around half of CYP with JIA [10–17]. Clinical features associated with disease severity or poor prognosis include ankle, wrist and/or temporomandibular joint arthritis, erosive disease, diagnostic delay, elevated inflammatory markers, and symmetric disease, and these may guide treatment escalation to biologic (b)DMARDs (often an anti-TNF) in combination with csDMARDs [8]. Anti-TNF agents that have demonstrated efficacy in JIA-related clinical trials include etanercept [18, 19], adalimumab [20, 21], golimumab [22, 23] and infliximab [24, 25], with adalimumab showing most success in refractory JIA-associated uveitis [26]. Despite some individuals having specific and similar disease phenotype, the heterogeneity of treatment response is not well understood. For instance, the anti-TNF agent most associated with ocular complications in individuals with ankylosing spondylitis is etanercept [27]. Cytokine imbalance may be a potential mechanism linking anti-TNF agents to ocular adverse events, but the underlying pathways remain unclear [28]. Moreover, the development of anti-drug antibodies to bDMARDs is common with variability across agents, and is associated with the inability to respond, inefficacy to treatment or disease recurrence [26, 29–34].

Rather than using the ‘one-size-fits-all’ approach, ‘precision medicine’ is centred around the concept of using genetic, cellular or molecular data to stratify individuals based on specific biological characteristics enabling better choice of targeted therapies. Precision medicine opportunities for different JIA subtypes have been previously reviewed [35]. Here, we discuss the approaches and technological advances in the molecular profiling of JIA, combined with analytical workflows and collaborative data platforms, which could enable further understanding of molecular mechanisms underpinning disease and tissue-based biomarkers of treatment response.

Despite the considerable expansion of therapeutic armamentarium, a substantial proportion of CYP experience persistent articular and extra-articular disease. Moreover, there are no validated biomarkers that reliably predict treatment response or reflect disease activity across subtypes. Characterizing synovial cellular, molecular and structural changes may identify robust biomarkers distinguishing those at heightened risk of rapid disease progression from those likely to follow a milder course. Furthermore, integrating detailed synovial pathology with advanced imaging modalities could help establish reliable correlates of tissue-level biomarkers. The identification of these non-invasive imaging signatures would support early disease stratification and longitudinal monitoring, potentially reducing the future need for invasive tissue sampling while maintaining diagnostic and prognostic accuracy.

## Strategies towards precision medicine in the treatment of JIA

Given the wide age range at disease onset and the potential for long-term therapy exposure, the development of a molecular taxonomy to individualize management and predict therapeutic response is a clinical priority. Such an approach could limit cumulative drug burden, unnecessary exposure to ineffective treatments, and the time to optimal disease control. The key challenges that must be addressed to achieve this goal are outlined below.

### Improving data accessibility

In a rare disease such as JIA and its associated uveitis, maximizing the utility of available data can be challenging. The Medical Research Council (MRC)’s CLUSTER (ChiLdhood arthritis and associated Uveitis: STratification through Endotypes and mechanisms to deliveR benefit) JIA Consortium (https://www.clusterconsortium.org.uk/) was formed to identify biomarkers of treatment response and prognostic indicators of JIA and JIA-associated uveitis [27]. CLUSTER has combined data from 5200 cases of JIA from several cohort studies and two clinical trials in JIA-associated uveitis, while working closely with patient and industry partners, leveraging multidisciplinary expertise in clinical and academic rheumatology, genetics, functional genomics, epidemiology, data sciences, immunology, deep phenotyping, statistics, bioinformatics and stratified medicine [28, 36–39]. Other UK initiatives include the MRC-funded TRICIA (Tissue Research in Childhood Inflammatory Arthritis) Consortium, which combines expertise in paediatric rheumatology, tissue analysis, data science and patient partnership for advances in precision medicine for CYP with JIA through studying high-quality synovial tissue (https://tricia.network/the-consortium/). There are wider bioresources to include all immune-mediated inflammatory disease (IMID), such as the IMID-Bio-UK biobanks (https://www.gla.ac.uk/research/az/imid/), which aim to unify existing biobanks to facilitate precision treatment of these conditions.

With increasingly large datasets, a major challenge comes with the vast information generated across various modalities. An international meeting of clinicians, trial experts, translational researchers and basic science researchers formed the London Declaration to improve care and ultimately cure childhood rheumatic disorders through worldwide collaboration [40], which included signatories from the CLUSTER consortium, PRINTO and UCAN (the Understanding Childhood Arthritis Network). UCAN is a large federation of networks focusing on translational research in childhood arthritis with a ‘hub and spoke model’ in the Netherlands, Singapore and Canada, which collates samples to be studied using computational biology and machine learning techniques to discover genetic, biological and phenotypic markers with diagnostic or prognostic potential in JIA (https://ucancandu.com) [41].

Obtaining sufficient biological samples to match the growing clinical data is an ongoing challenge, reflecting the mismatch between the healthcare setting and research needs. Batch effects across different datasets can make meta-analyses challenging. Combining data across studies may be achieved through imputation, which involves replacing missing data with approximates to minimize bias and achieve power in statistical analyses. However, this process is complex and time-consuming as demonstrated through efforts of combining four different JIA outcome studies [36]. An important way to support data integration is to prepare a core set list agreed by experts internationally and collect samples during clinical care, as illustrated in the CAPTURE [Consensus-derived, Accessible (information), Patient-focused, Team-Focused, Universally-collected (UK), Relevant to all and containing Essential data items] JIA dataset [42] and other rare paediatric IMID such as juvenile dermatomyositis [43]. The rising use of a few platforms for electronic health records may make data capture more feasible in future. For clinical trials, established support is available for pharmacokinetic and pharmacodynamic analyses, but opportunities for biomarker and mechanistic studies remain limited.

The harmonization of large datasets would enable reproducibility of multicentre data [41] to improve data accessibility and minimize loss according to the FAIR (findable, accessible, interoperable and reusable) principles [44]. Ideally data would be standardized and stored in a user-friendly platform. This democratization of access in repositories and training resources would enable the data to be available to the public, individuals with JIA, healthcare professionals and researchers to use. Research governance would need to be considered to protect and audit the use of sensitive data. Together, these efforts would enable individuals of varying technical ability to freely access the data, enabling a holistic approach to improving precision medicine for JIA.

### Cellular and molecular phenotyping

Efforts to dissect the cellular and molecular mechanisms underpinning the diverse clinical phenotypes in JIA are ongoing, opening important avenues for the development of precision medicine approaches. Increasingly sophisticated methodologies have enabled the detailed interrogation of immune cell ontogeny, differentiation states and functional phenotypes, and characterization of tissue-resident stromal and immune cells within inflamed synovium. The application of high-dimensional transcriptomic technologies and advanced cellular profiling platforms has facilitated the identification of molecular signatures and pathogenic cell subsets that may drive disease heterogeneity. These approaches provide critical insight into the immunopathogenic architecture of JIA and offer a framework for biomarker discovery and mechanism-based therapeutic stratification.

Most studies have focused on a selection of molecular markers or cell type, in blood or synovial fluids obtained at therapeutic aspiration, which may restrict the global or spatial study of cell–cell regulatory networks. Tissue-resident immune cells represent a small proportion of the total cells in inflamed tissues, limiting their characterization by transcriptional profiles. In the joint, this issue is challenging given the limited availability of samples for study, since very few studies previously obtained synovial tissue for research use from children with JIA. However, this research gap has recently started to close with the launch of a large tissue-based study of the synovia in JIA [45]. This advance is of crucial relevance, as in JIA and most IMIDs, there is local or systemic activation of the immune system. For example, in the synovia of patients with rheumatoid arthritis, immune cells that regulate both inflammation and remission have been discovered [46–49], however it is not known if the markers in the synovial fluid may reflect that of tissue.

The S100 proteins S100A8/9 (also known as MRP8/14 or calprotectin) and S100A12 are proinflammatory proteins that act as ligands of Toll-like receptor 4 and phagocyte activation markers [50–52]. Serum levels of S100A8/9 correlate with disease activity and can differentiate patients who respond well to methotrexate [53, 54] as well as predict flares upon drug withdrawal [13]. Serum S100A12 protein concentrations correlated with treatment response in individuals with JIA taking methotrexate and anti-TNF [55]. The measurement of S100A12 with CRP during treatment withdrawal in individuals with JIA predicted flares better than S100A8/9 alone [56]. Elevated levels of serum S100A8/9 and S100A12 in CYPs with JIA-associated uveitis may reflect intraocular disease [54]. These biomarkers reflect inflammation but may lack specificity in JIA or JIA-associated uveitis.

Interleukin and chemokine levels have been considered as potential biomarkers of disease activity although their relative instability in clinical samples would make them challenging to translate to clinical use. Individuals with polyarticular JIA have higher IL-33 (mediates Th2 pathways) concentrations compared with oligoarticular JIA and healthy controls, and increased IL-33 correlates with RF-positive disease [57]. Plasma from individuals with oligoarticular and polyarticular JIA had increased IL-17, but not in systemic JIA [58]. In matched blood and synovial fluid of individuals with oligoarticular, polyarticular and systemic JIA, higher levels of IL-6, IL-15, CCL2, CCL3, CXCL8, CXCL9 and CXCL10 were observed in synovial fluid [58]. Levels of synovial IL-17 correlated with the number of swollen, restricted and painful joints in individuals with JIA, and IL-17 induced IL-6, IL-8, MMP1 and MMP3 production by synovial fibroblasts in the presence of TNF and IL-17 [59].

Synovial tissue provides an important resource for studying cellular profiles directly from the site of inflammation, some of which are not detectable in blood. Whether synovial fluid is a robust surrogate for tissue biology in JIA remains to be determined. The synovial fluid monocytes from individuals with oligoarticular JIA had increased expression of surface markers CD40, CD86 and CD206, but not CD163, compared with paired blood monocytes [60], indicating potential inflammation. Synovial regulatory T cells (Tregs) are phenotypically different from healthy Tregs [61]. A subset of Tregs express CD161, which marks their potential for proinflammatory cytokine production [62]. The TCRβ repertoires of CD161^+^ and CD161^−^ Tregs and CD161^+^ and CD161^−^ T conventional cells showed overlap in transcriptional and protein signatures from inflamed sites in autoimmune arthritis, but not in the blood, suggesting the possibility of CD161 having a role in pathogenesis [63] and potential Treg plasticity at the inflamed site. Blood Treg signature and subsets may serve as useful biomarkers to differentiate disease progression and remission in JIA. In a study that used machine learning to generate a model to distinguish Tregs in active JIA from healthy controls, individuals with active JIA had TIGIT^high^CD226^high^CD25^low^ Teff-like Tregs, whereas those with inactive JIA had CD39^-^TNFR2^-^Helios^high^ and 4-1BB^low^TIGIT^low^ID2^intermediate^ Tregs [64]. Additionally, individuals with JIA had activated synovial fluid T effector and Tregs, with functions suggestive of metabolic reprogramming via the expression of CD71, TNFR2 and PD-1 [65]. The synovial fluid effectors largely consist of the CD4^−^Foxp3^+^ T cells in the inflamed joint, and blood CD4^+^Foxp3^+^ Tregs are increased individuals with active but not inactive JIA, highlighting differences in cellular phenotype in the circulation and distally inflamed sites [65]. Single-cell analyses of synovial fluid from patients with oligoarticular JIA reveal heterogeneous effector and regulatory subsets, including IFN-induced Tregs, peripheral T helper cells and cytotoxic CD4^+^ T cells, and transcriptomic profiling confirm a Th1 signature in the synovial CD4^+^ T cells, different from the profile of circulating cells [66]. Single-cell and epigenetic profiling identify a unique Treg profile involving the upregulation of core regulatory markers (*FOXP3, CTLA4, TIGIT*) and genes associated with broader effector characteristics (*GITR*, *BLIMP-1*, *BATF*) in the inflamed joint [67]. Together, Treg phenotypic cells may serve as potential biomarkers to assess disease activity and management to sustain remission.

### Multimodal data and machine learning for JIA

**Figure 1:**
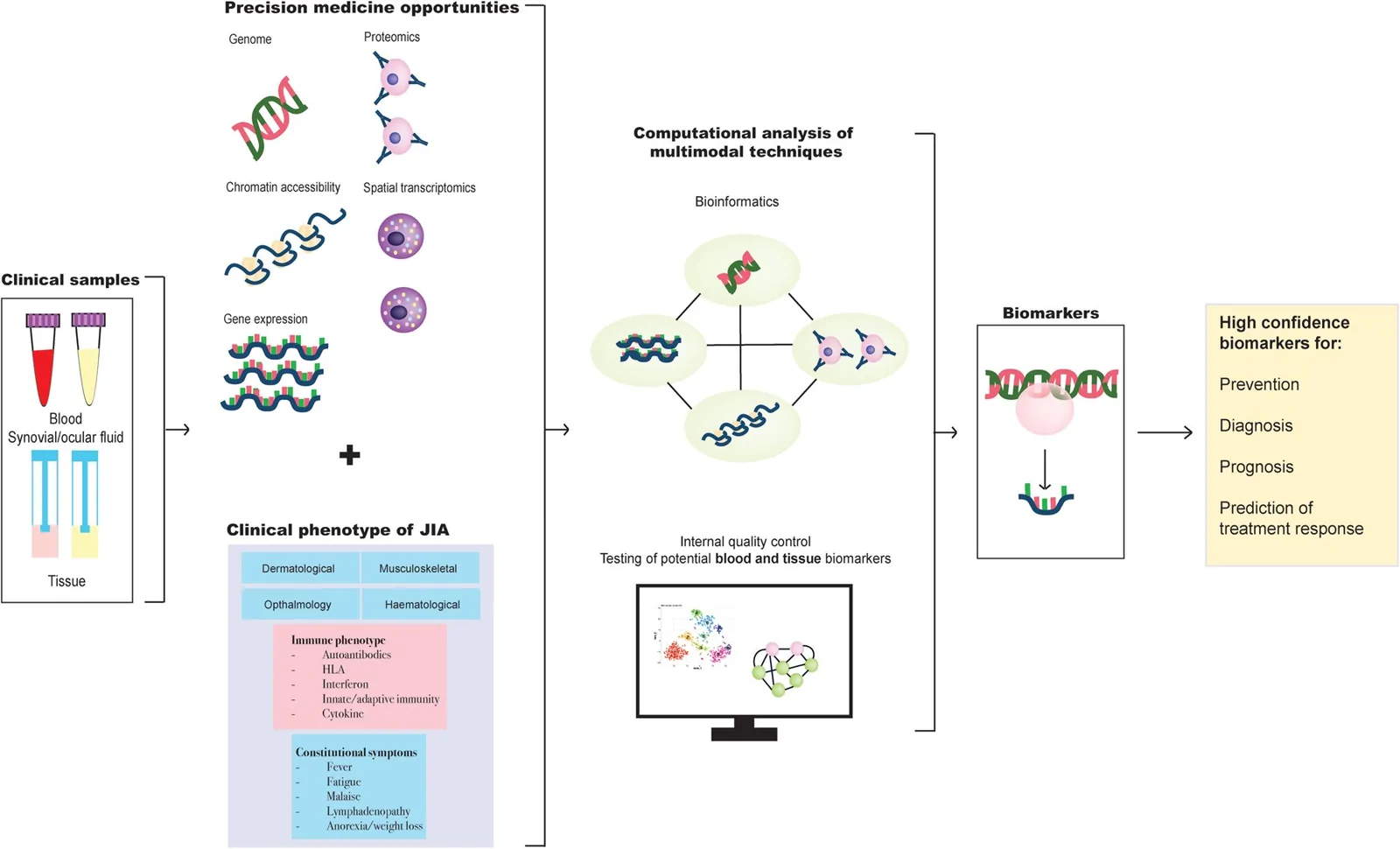
Schematic illustration of the integration and validation of high dimensional data derived from blood and tissue alongside clinical phenotype to detect high confidence biomarkers that can be used in the clinical setting and trials in JIA. HLA: human leucocyte antigen; JIA: juvenile idiopathic arthritis

Single-level technologies include flow cytometry, mass cytometry, transcriptomics and functional assays, which have enabled further characterization of novel immune cell types. To dissect complex gene-regulatory and cell crosstalk networks, studying multiple modalities of the genetic and phenotypic cellular responses that dictate function could be crucial. The development of precision medicine for individuals with JIA could benefit from such an integrative approach, in which two or more omics datasets of molecules that make up cells, including DNA, chromatin, genes and proteins, are combined alongside clinical data (Fig. 1).

While single-cell RNA-sequencing has enabled regulatory networks such as Th1 phenotypes and disease severity to be determined in patients with JIA [66], these processes are challenging to interpret from the transcriptome alone. Gaining access to DNA or chromatin through single-cell ATAC-seq will help to determine functional genomic profiles that dictate cellular state [68]. Single-cell ATAC-seq, when used alongside transcriptomic methods, may identify and map transcription factor binding sites that potentially control specific genes. An example of the application of this multi-omics approach showed changes in gene expression and chromatin accessibility in the peripheral CD4^+^ T cells of individuals with active JIA compared with healthy controls, implying a global immune dysfunction rather than disease affecting the joints alone [69]. Proteins direct gene regulatory networks, and protein abundance can be measured through proteomic methods, which enables intercellular and intracellular signalling pathways to be mapped out. The study of RNA and protein epitopes may infer the possibility of post-transcriptional modifications that if dysregulated could serve as potential therapeutic targets. Complex bioinformatics are required to detect biologically relevant pathways within multimodal techniques, which could be linked to JIA. Computational tools to validate potential biomarkers through integrating existing databases would enable the reproducibility and translational utility of these candidates in the clinic. Advances in spatial technologies would enable the construction and visualization of whole transcriptome three-dimensional atlases at single-cell resolution. Recent work utilizing multimodal techniques including single-cell RNA-seq, multiplex immunofluorescence and spatial transcriptomics has generated a synovial tissue atlas in JIA, assessed gene signatures predictive of future severity, and compared JIA to adult RA synovium at high resolution. This work has revealed spatial tissue niches with both immune and stromal cell populations, with associated genes linked to disease severity and risk to effector cell populations, including tissue resident SPP1^+^ macrophages and fibrin-associated myeloid cells [45].

Major hurdles in multimodal approaches include the expertise to resolve the heterogeneity across various datasets, methods to address missing or unknown data, unmatched sample pairs, and variable processing techniques used on different cells or tissues. The integration with other data modalities is crucial for improved cellular and tissue characterization. Machine learning or statistical learning is one of the ways to improve the interpretability of data and attempts to approximate humans’ ability to objectively recognize patterns using computation [70]. Machine learning supersedes conventional regression model approaches by capturing more complex, non-linear relationships observed in clinical and multimodal data. For instance, machine learning analysing clinical trajectory data has identified six distinct JIA subtypes post-methotrexate initiation [37] and rank single-nucleotide polymorphisms (SNP)–gene–tissue contributions to JIA risk [71], which may inform the heritability of JIA risk and how we treat early disease. The missingness of information from datasets may be addressed through supervised or unsupervised learning [72]. It is uncertain whether imputation through supervised learning could accurately infer how cell crosstalk networks function across cells and tissues in health and disease, or whether modelling causality in such a way could introduce bias when characterizing patients’ disease profiles prior to treating their arthritis.

## Outlook for precision medicine in JIA

Multimodal studies have generated a breadth of data that provide insight into the molecular biology of disease, but there are challenges in validating such discoveries. While the data from within a specific tissular niche or environment, for instance synovial fluid or tissue in JIA, have been informative in view of potential biological mechanisms, the extent of transcripts generated have been difficult to compress into a biomarker fit for clinical use. Proteomics may be particularly challenging in tissue samples due to difficulties in the detection of proteins of low abundance that may be of clinical significance, lack of signal correlation with immune cells relevant to disease, and limited samples. Synovial biopsies are currently performed in very few centres in the world and may therefore lack practicality. Ideally, transcriptional and protein profiles need be integrated with the blood, which could indicate downstream signals. However, the limitations in depending on blood biomarkers is the extreme range of RNA or protein expression, the masking of low abundance transcripts or peptides, and the lack of correlation with tissue profiles. Spatial transcriptomics may enable some congruity or validation of biomarkers across datasets. Thus, we are still far from a single biomarker or a panel of biomarkers that could be easily obtained and affordable across centres to predict or prognose individuals with JIA, and there are further critical steps towards biomarker validation and therapeutic stratification (Box 1).

Box 1 Questions to validate discoveries from JIA subtype studies in view of precision medicine.

Could multimodal data be used to identify disease biomarkers including spatial cell regulatory networks and cellular subtypes with clinical utility in JIA?

Could we identify correlates of inflamed tissue pathology in accessible anatomical compartments including blood and synovial fluid for longitudinal sampling to monitor disease activity and maintain remission in JIA?

Could we use machine learning to explore complex and integrated datasets to improve our understanding of biological pathways in JIA?

How do we move from high resolution technologies at a single cell level to low resolution technologies to determine cell regulatory networks of biological relevance in JIA?

Computational strategies that integrate these distinct data layers, such as joint similarity matrix learning, allow combined analysis of cellular relationships across modalities [73]. This enables visualization (e.g. dimensionality reduction), clustering and trajectory modelling to identify pathogenic cell subsets, transitional cellular states and regulatory networks that may not be apparent using single-platform analyses. Clinically, such approaches could help define molecular endotypes that cut across traditional phenotype-based classifications, for example, distinguishing children at risk of persistent synovitis, joint damage or extra-articular complications from those likely to achieve sustained remission.

To maximize clinical impact, robust experimental metadata and harmonized, well-annotated clinical datasets must accompany molecular data [28]. Open data sharing and coordinated validation studies will be essential to ensure reproducibility and enable meta-analyses across cohorts. Ultimately, the goal is the development of reproducible, high-confidence biomarkers with clear translational utility, tools that clinicians can use in routine practice to define disease state, predict prognosis, and select the most appropriate therapy at the earliest opportunity.

The future of paediatric rheumatology, including for those living with JIA, is advancing. Progress is being driven by large-scale clinical and epidemiological datasets, genetic and multi-omic research, tissue atlas initiatives, and strengthened international collaboration, alongside meaningful engagement of patients, families, and industry partners. Although significant analytical and logistical challenges remain, advances in multimodal technologies and computational methodologies are bringing precision medicine for CYP with arthritis increasingly within reach.

## Search strategy

References were identified through Medline and PubMed with terms ‘juvenile idiopath* arthrit*’ or ‘juvenile idiopathic arthritis’ or ‘JIA’ AND ‘multi-omic*’ or ‘genomics’ or ‘proteomics’ or ‘metabolomics’ or ‘transcriptomics’ or ‘epigenomics’. For methodological approaches, papers from the last 5–10 years were selected. The broader reference list was selected based on relevance to the wider scope of our review.

## Data Availability

No new data were generated or analysed in support of this review article.
